# Feasibility of a Fully Covered Self-Expandable Metal Stent with a Central Waist and a Long Removal String for Management of Malignant Distal Biliary Obstruction: A Multicenter Prospective Pilot Study

**DOI:** 10.3390/jcm15145612

**Published:** 2026-07-17

**Authors:** Min Jae Yang, Dong Wook Lee, Dong Kee Jang, Kwang Bum Cho, Seok Jeong, Sung Ill Jang

**Affiliations:** 1 Department of Gastroenterology, Ajou University School of Medicine, Suwon 16499, Republic of Korea; creator1999@hanmail.net; 2 Department of Internal Medicine, School of Medicine, Kyungpook National University, Daegu 41404, Republic of Korea; storm5333@naver.com; 3 Department of Medicine, Samsung Medical Center, Sungkyunkwan University School of Medicine, Seoul 06351, Republic of Korea; mapmotive@hanmail.net; 4 Department of Internal Medicine, Keimyung University Dongsan Medical Center, Keimyung University School of Medicine, Daegu 1035, Republic of Korea; chokb@dsmc.or.kr; 5 Department of Internal Medicine, Inha University Hospital, Inha University College of Medicine, 27 Inhang-ro, Jung-gu, Incheon 22332, Republic of Korea; 6 Department of Internal Medicine, Gangnam Severance Hospital, Yonsei University College of Medicine, 712 Eonjuro, Gangnam-gu, Seoul 06273, Republic of Korea

**Keywords:** malignant biliary obstruction, biliary stricture, self-expandable metal stent, pancreatic cancer, bile duct cancer, stent migration

## Abstract

**Background/Objectives**: A fully covered self-expandable metal stent (FCSEMS) with a central waist and long retrieval string has recently been introduced for benign biliary strictures; however, its role in malignant biliary obstruction (MBO) remains unclear. This prospective, multicenter study evaluated the feasibility of palliation for unresectable distal MBO. **Methods**: Between April 2022 and March 2023, patients with extrahepatic MBO were enrolled across six tertiary referral centers. The FCSEMS was a silicone-covered, cross-wired nitinol stent with an anti-migration central waist 2 mm narrower than both ends, three radiopaque markers, and a 10 cm retrieval string. Stent-related outcomes and adverse events were analyzed. **Results**: Both technical and clinical success rates were 100% (46/46). The median stent patency and survival times were 251 and 360 days, respectively. Stent dysfunction occurred in 18 patients (39.1%), including 10 with migration and 8 with occlusion. Stent migration was observed exclusively in the transpapillary stenting group and showed no significant association with stricture length, stent-bending angle, or the ratio of stent lengths above and below the maximal bending point. All the occluded stents were successfully removed. Early non-recurrent biliary obstruction (RBO) adverse events occurred in five patients (three with pancreatitis, one with bleeding, one with cholecystitis), whereas late non-RBO events were reported in three patients (two with cholecystitis, one with cholangitis). **Conclusions**: An FCSEMS with a central waist and retrieval string is feasible for the palliation of unresectable distal MBO. Optimization of the mechanical properties is warranted to minimize migration.

## 1. Introduction

Endoscopic placement of self-expandable metal stents (SEMSs) is the primary palliative treatment for unresectable distal malignant biliary obstruction (MBO) [1]. Their larger diameter allows SEMSs to offer superior patency over plastic stents by preventing biliary sludge accumulation [2,3]. The emergence of new therapeutic options has extended the survival time to nearly one year for patients with metastatic pancreatobiliary cancer, creating an urgent need for innovative SEMS designs that maintain long-term patency throughout the patient’s life span.

The bare metal mesh structure of uncovered SEMSs facilitates stent stability through tissue embedding but is prone to tumor ingrowth. Although synthetic coverings counteract ingrowth, challenges persist with tumor overgrowth, risk of stent migration, and biliary sludge retention [4,5,6,7]. Consequently, neither uncovered nor covered SEMSs consistently outperform the others in stent patency [4,5,8,9]. However, fully covered SEMSs (FCSEMSs) are increasingly preferred in clinical practice as they facilitate easier stent exchange during reintervention and can enhance the patency of a second stent after reintervention compared with uncovered SEMSs [10,11].

An FCSEMS with a central waist and long retrieval string (Kaffes; Taewoong Medical Co., Ltd., Goyang, Republic of Korea) was originally designed for intraductal placement to treat benign biliary strictures [12,13]. The central waist increases radial force and enables selective short coverage of the stricture site, thereby preventing migration, whereas a long retrieval string facilitates stent removal. It has shown high efficacy in treating anastomotic biliary strictures after liver transplantation, as well as non-transplantation benign biliary strictures [13,14]. However, its performance for MBO has not yet been validated. This pilot study evaluated the feasibility of using FCSEMSs for durable palliation of patients with unresectable distal MBO.

## 2. Materials and Methods

### 2.1. Patients

Between April 2022 and March 2023, patients with unresectable distal MBO were prospectively enrolled across six tertiary referral centers. The inclusion criteria were as follows: (1) age > 20 years; (2) MBO located >2 cm distal to the hepatic hilum; and (3) ineligibility for curative surgery due to metastasis, locally advanced disease, or high surgical risk. Exclusion criteria were as follows: (1) coagulopathy characterized by an international normalized ratio (INR) > 1.5 or platelet count < 50,000); (2) American Society of Anesthesiologists physical status grade IV; and (3) inability to provide informed consent. This prospective pilot study was approved by the Institutional Review Board of each participating hospital. Written informed consent was obtained from all participating patients prior to enrollment. Additionally, this prospective study was registered with the Clinical Research Information Service under the registration number KCT0007263.

### 2.2. Stent Profile and Endoscopic Procedure

**Figure 1:**
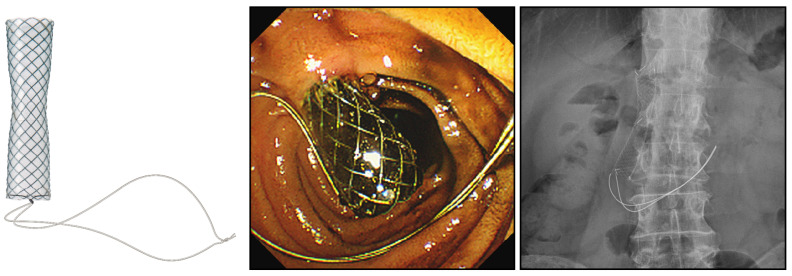
The fully covered self-expandable metal stent with a central waist and a long retrieval string (Kaffes; Taewoong Medical Co., Ltd., Goyang, Republic of Korea).

The Kaffes stent is a silicone-covered metal stent constructed from a nitinol-based crosswire mesh (Figure 1). It incorporates a 2 mm gradual narrowing at the center to reduce the risk of migration and enhance the radial force at the stricture site. Employing a minimum-length stent with the central waist precisely positioned across the stricture avoided unnecessary pressure on the normal bile duct. A 10 cm platinum radiopaque retrieval string facilitated stent removal, while three radiopaque markers, located at both ends and the midpoint, enabled accurate fluoroscopic identification. The stents used in this study had diameters of 6, 8, and 10 mm at either end and lengths of 4, 5, 6, 7, and 8 cm. The introducer diameter was 8.5 Fr for the 6-mm and 8-mm sizes, and 9 Fr for the 10-mm size.

Endoscopic retrograde cholangiopancreatography (ERCP) was performed using a side-viewing duodenoscope (JF-260V or TJF-260V; Olympus Corp., Tokyo, Japan) under conscious sedation with standard doses of midazolam, propofol, and meperidine. Stent length was determined according to the longitudinal location of the stricture and the anticipated safety margin. The choice between suprapapillary and transpapillary stent placement was determined at the discretion of the attending operator based on a comprehensive evaluation of preprocedural cross-sectional imaging and intraoperative fluoroscopic findings. Specifically, suprapapillary placement was preferentially attempted based on the following anatomical, procedural, and clinical factors: (1) a stricture location sufficiently distant from the papilla with a stricture length that allowed for a stable proximal margin, (2) a sufficient distance from the papilla to the stricture to obtain an adequate distal safety margin above the sphincter of Oddi, (3) a predictable, less angulated bile duct configuration that minimized the risk of stent migration, and (4) a high patient risk profile for post-ERCP pancreatitis.

### 2.3. Outcome Measurements

In this pilot study, the feasibility of the Kaffes stent was operationally defined as achieving a technical success rate of greater than 90% and the absence of fatal procedure-related complications within 30 days. While technical success, clinical success, and successful removability are key components of device feasibility, they have become highly standardized with contemporary FCSEMSs and thus offer limited clinical differentiation among different stent designs. Consequently, cumulative stent patency was designated as the primary outcome to serve as a preliminary efficacy indicator, aimed at providing the necessary pilot data for sample size calculation in a future definitive randomized controlled trial.

Adverse events were defined and graded according to the American Society for Gastrointestinal Endoscopy Severity Grading system [15] and Tokyo Guidelines [16,17]. Clinical success was defined as a decrease in serum total bilirubin levels to less than half of the pretreatment baseline or its normalization within 2 weeks following stent placement, without the need for immediate re-intervention. Stent patency was defined as the interval between the date of stent insertion and the date of documented stent dysfunction, or the date of death if no dysfunction occurred during the patient’s lifetime. Stent dysfunction comprised stent occlusion and migration leading to recurrent biliary obstruction, which was clinically defined as the presence of biochemical evidence of cholestasis accompanied by biliary dilation on imaging studies [18].

**Figure 2:**
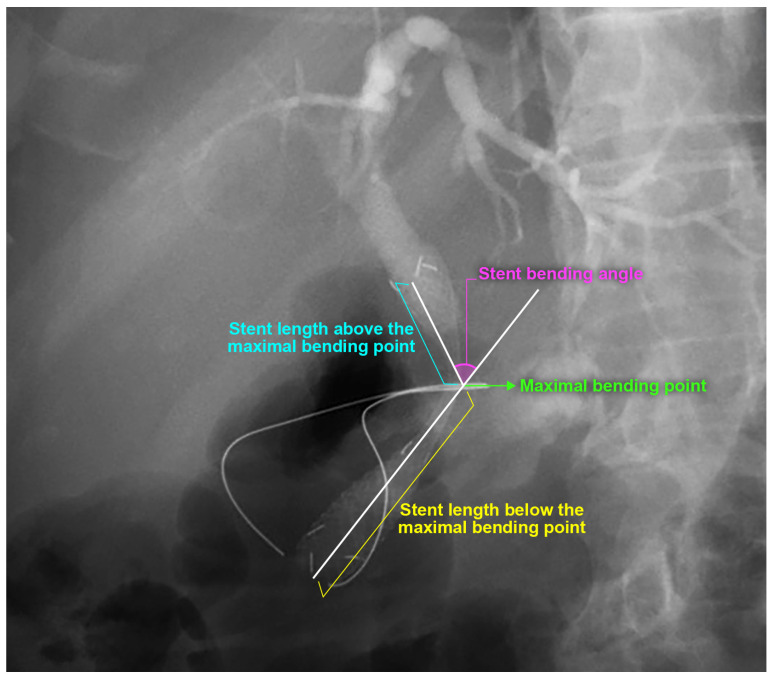
Stent position-related variables influencing axial force: the stent bending angle and the ratio of stent lengths above and below the maximal bending point.

To evaluate the potential determinants of stent migration, Stent position–related variables potentially influencing axial force were analyzed, including the ratio of stent-to-stricture length, stent bending angle, and ratio of stent lengths above and below the maximal bending point (Figure 2). The maximal bending point was defined as the site of greatest curvature of the stent within the bile duct immediately after placement. The stent bending angle was defined as the angle between the portion of the stent above the maximal bending point and the straight line parallel to the portion below it.

### 2.4. Statistical Analyses

The target sample size was chosen empirically based on practical recruitment feasibility across the participating referral centers, which is considered appropriate for exploratory pilot trials aimed at generating baseline data for future large-scale, definitive comparative studies. Categorical variables were analyzed using the chi-square test or Fisher’s exact test, as appropriate. Differences in quantitative data were assessed using an unpaired *t*-test and are presented as mean values. Stent patency and patient survival were evaluated using the Kaplan–Meier method. To account for death as a competing risk, the cumulative incidence function for stent dysfunction was estimated. Competing-risk analyses were performed using R statistical software (version 4.5.2; R Foundation for Statistical Computing, Vienna, Austria), whereas all other statistical analyses were conducted using SPSS software (version 25.0; IBM Corp., Armonk, NY, USA). Statistical significance was set at *p* < 0.05.

## 3. Results

Baseline patient characteristics are summarized in Table 1. A total of 46 patients were included in this study. The mean age was 73.8 years, and the cohort comprised 24 males and 22 females. Prior biliary drainage was performed in nine patients (19.6%), and the average stricture length was 27.1 mm. Pancreatic cancer was the most common diagnosis (28 patients, 60.9%) followed by bile duct cancer.

The stent-related outcomes are presented in Table 2. Both technical and clinical success rates were 100% (46/46). During a median follow-up of 225 days, stent dysfunction occurred in 18 patients (39.1%), including 10 with stent migration and 8 with stent occlusion due to biliary sludge (*n* = 7) or tumor overgrowth (*n* = 1). No cases of stent obstruction due to tumor ingrowth were observed. Fourteen patients underwent reintervention, consisting of 12 endoscopic procedures and 2 percutaneous transhepatic biliary drainages. Stent removal was successful in all patients with stent occlusion.

**Figure 3:**
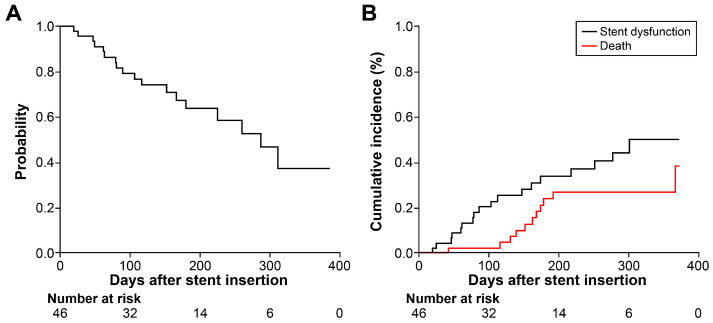
Cumulative stent patency and cumulative incidence of stent dysfunction with death. (**A**) Kaplan–Meier curve demonstrating the cumulative stent patency over time, where death was treated as a conventional censoring event. (**B**) Cumulative incidence function curves for stent dysfunction (black solid line) and death (red solid line) analyzed under a competing risk framework, treating death before stent dysfunction as a competing risk.

The median cumulative stent patency based on Kaplan–Meier analysis was 251 days (interquartile range, 207–287 days). The patency rates at 3, 6, and 12 months were 79.2%, 63.8%, and 56.0%, respectively (Figure 3A). However, because death prior to stent dysfunction represents a significant competing risk in this patient population, a competing risk survival analysis was further performed (Figure 3B). When evaluating the cumulative incidence function, the cumulative incidence of stent dysfunction was 34% at 6 months and 50% at 12 months, while the cumulative incidence of death as a competing risk was 21% and 27% at the respective time points. There was no significant difference in stent patency between the suprapapillary and transpapillary groups (311 days vs. 260 days, *p* = 0.648). Adverse events occurred in 8 (17.4%) patients in Table 3. The incidence of adverse events, including pancreatitis, did not significantly differ between the suprapapillary and transpapillary groups. Notably, stent migration occurred only in the transpapillary group (26.3% vs. 0%), though this numerical difference did not reach statistical significance (*p* = 0.171).

To explore the potential physical determinants associated with stent migration, we analyzed variables reflecting the spatial relationship between the stent and stricture as well as between the stent and the maximal bile duct bending point, potentially affecting axial force (Table 4). However, no statistically significant differences were found between the migration and non-migration groups in terms of the stent-to-stricture length ratio, stent bending angle, or ratio of stent lengths above and below the maximal bending point.

## 4. Discussion

This multicenter prospective study is the first to evaluate the feasibility of an FCSEMS with a central waist and long retrieval string, originally designed for intraductal use in benign biliary strictures, in patients with distal MBO. Stent-related outcomes were comparable to those reported in previous studies; however, stent migration was observed more frequently with transpapillary placement than with suprapapillary placement, although the difference did not reach statistical significance.

Recent studies have reported favorable outcomes in terms of stent patency when FCSEMSs are placed in a suprapapillary fashion for unresectable MBO [19]. These outcomes can be attributed to reduced formation of biliary sludge, which is likely the result of decreased duodenobiliary reflux. In addition, as intraductal stents are placed as short as possible to achieve a balanced position across the stricture, and the tapered distal common bile duct acts as a barrier against distal displacement, stent migration is rarely observed. In this study, no stent migration was observed in the suprapapillary group, as expected. However, with respect to biliary sludge formation, suprapapillary placement did not demonstrate clear superiority over transpapillary placement, and the overall stent patency showed no statistically significant differences between the two groups. However, given the small sample size in the suprapapillary group, further large-scale comparative studies are warranted.

In this study, the risk of stent migration associated with transpapillary placement remained a considerable challenge. According to a recently published meta-analysis, the pooled rates of recurrent biliary obstruction and stent migration for conventional FCSEMSs were reported to be 27.3% and 9.8%, respectively [20]. In our study, the RBO rate was higher at 39.1%, which was primarily attributed to a higher stent migration rate of 21.7% in the transpapillary group. The stent was originally designed to cover benign biliary strictures. Therefore, it prioritizes radial force for effective stricture expansion through a cross-knit braided mesh structure composed of a thick wire [21]. As a tradeoff, the axial force was increased, which led to reduced conformability and a higher incidence of stent migration during transpapillary applications. The central waist of the stent enhances the radial force and enables selective coverage of the stricture site in a short, saddle-like fashion with intraductal placement, thereby preventing migration. However, these advantages are diminished in the context of transpapillary application using a longer stent length, as the eccentric alignment between the waist and malignant stricture reduces its stabilizing effect. Consequently, the anti-migration benefit of the central waist was offset by the pro-migration tendency associated with the increased axial force.

In this study, we sought to identify potential physical determinants associated with stent migration by analyzing the stent bending angle and the correlation between the maximal bending point and stent position in patients with and without stent migration. This is because the axial force increases with greater angulation within the bile duct and shorter stent length above the bending point of the stent [22,23]. In the migration group, the stent angle tended to be steeper with a shorter proximal stent length relative to the maximal bending point, resulting in a smaller ratio of stent lengths above and below the maximal bending point. However, no statistically significant differences were observed between the migratory and non-migratory groups. Due to the small sample size and potential interobserver variability, these findings should be interpreted strictly as exploratory, hypothesis-generating clues; therefore, future prospective studies involving larger cohorts are warranted to assess the clinical relevance of these underexplored physical factors that may augment the axial force.

Regarding procedure-related complications, previous literature comparing FCSEMSs with uncovered SEMSs reported post-ERCP pancreatitis rates ranging from 0% to 13.6% and acute cholecystitis rates from 0% to 15.3% [24]. Both our pancreatitis (6.5%) and cholecystitis (6.5%) rates in this study fall within these documented ranges.

In patients with MBO who have the gallbladder in situ, the use of covered SEMSs has raised concerns regarding cholecystitis, primarily due to cystic duct obstruction from the covering membrane, resulting in a jailing effect [25]. In the present study, although the axial force was increased, the incidence of cholecystitis was not higher than that reported in previous studies. We hypothesize that the concave configuration of the central waist may help mitigate the cystic duct jailing effect.

Despite being a multicenter prospective study, this research has the limitations of an overall small sample size and an imbalance between the suprapapillary and transpapillary groups, which inevitably resulted in underpowered study outcomes. Furthermore, the single-arm design and lack of comparison with other FCSEMS groups limits the generalizability of the study findings. Additionally, the study population included a heterogeneous mix of underlying malignancies. Because each disease entity possesses distinct tumor biology, stricture morphology, and progression patterns, this clinical heterogeneity may have influenced stent patency and dysfunction behavior. However, a formal stratified subgroup analysis was not statistically feasible due to the small sample size and limited number of events within each etiology. Future large-scale, disease-specific trials are required to determine how individual tumor characteristics impact long-term stent performance. Another limitation is that the median follow-up duration was 225 days, which is shorter than the 12-month landmark timeline. Consequently, the 12-month stent patency rate and long-term survival estimates involve statistical extrapolation and possess limited maturity owing to the small number of patients remaining at the tail end of the follow-up period.

## 5. Conclusions

This multicenter pilot study demonstrated that the novel FCSEMS with a central waist and extended retrieval string provides a feasible therapeutic option for unresectable distal MBO, yielding high success rates. However, stent migration remains a significant limitation. Given that the difference in migration rates between the positioning groups did not reach statistical significance due to the limited sample size, further large-scale prospective studies are warranted to clarify the definitive relationship between stent positioning and migration risk.

## Figures and Tables

**Table 1:** Baseline characteristics of patients.

	*n* = 46
Age, year	73.8 ± 10.2
Sex (male/female), *n*	24/22
Prior biliary drainage, *n* (%)	9 (19.6)
History of cholecystectomy, *n* (%)	3 (6.5)
Bilirubin level on admission, mg/dL	7.1 ± 6.8
Stricture length, mm	27.1 ± 7.8
Diagnosis, *n* (%)	
Pancreatic cancer	28 (60.9)
Bile duct cancer	13 (28.3)
Gallbladder cancer	3 (6.5)
Ampullary cancer	2 (4.3)
Palliative CTx and/or RTx, *n* (%)	19 (41.3)

Continuous variables are expressed as mean ± standard deviation. Abbreviations: CTx = Chemotherapy; RTx = Radiotherapy.

**Table 2:** Main outcomes of the study (*n* = 46).

Technical success	46 (100)
Clinical success	46 (100)
Follow-up period, median, days	225
Stent dysfunction, *n* (%)	18 (39.1)
Causes of stent dysfunction, (%)	
Stent distal migration	10
Biliary sludge	7
Tumor overgrowth	1
Reintervention, endoscopic/percutaneous	12/2
Cumulative stent patency ^1^	251 (207–287)
3-M/6-M/12-M stent patency rate, %	79.2/63.8/56.0
Patient survival ^1^	360 (293–426)

^1^ Analyzed using the Kaplan–Meier method and log-rank test, expressed as estimated median values with interquartile ranges.

**Table 3:** Adverse events, including stent dysfunction.

	Total (*n* = 46)	Supra-Papilla (*n* = 8)	Trans-Papilla (*n* = 38)	*p* Value
Overall adverse events ^1^ *n* (%)	8 (17.4)	1	7	1.000
Pancreatitis, *n* (%)	3 (6.5)	1	2	0.444
Bleeding, *n* (%)	1 (2.2)	0	1	1.000
Cholecystitis, *n* (%)	3 (6.5)	0	3	1.000
Cholangitis without stent dys function, *n* (%)	1 (2.2)	0	1	1.000
Stent dysfunction *n* (%)	18 (39.1)	3	15	1.000
Migration	10 (21.7)	0	10	0.171
Occlusion	8 (17.4)	3	5	0.129

^1^ More than one adverse event per patient was considered as one event.

**Table 4:** Potential factors associated with stent migration in the transpapillary stenting group.

	Migration (*n* = 10)	Non-Migration (*n* = 28)	*p* Value
Stricture length	30.1	27.7	0.488
Ratio of stent and stricture length	2.3	2.0	0.322
Stent bending angle, degree	27.6	23.4	0.564
Stent length above the maximal bending point	21.0	24.6	0.280
Stent length below the maximal bending point	30.5	31.2	0.818
Ratio of stent lengths above and below the maximal bending point	0.75	0.85	0.609

Length-related variables are expressed as mean values in mm.

## Data Availability

Data are available upon reasonable request.

## Abbreviations

The following abbreviations are used in this manuscript:

SEMS	Self-expandable metal stent
MBO	Malignant biliary obstruction
FCSEMS	Fully covered self-expandable metal stent
ERCP	Endoscopic retrograde cholangiopancreatography
RBO	Recurrent biliary obstruction
